# Repair of Postoperative Abdominal Hernia in a Child with Congenital Omphalocele Using Porcine Dermal Matrix

**DOI:** 10.1155/2016/1828751

**Published:** 2016-03-24

**Authors:** V. Lambropoulos, E. Mylona, V. Mouravas, C. Tsakalidis, I. Spyridakis, G. Mitsiakos, P. Karagianni

**Affiliations:** ^1^Pediatric Surgery Clinic, General Hospital Papageorgiou, Ring Road N. Efkarpia, 56429 Thessaloniki, Greece; ^2^Neonatal Department, Aristotle University of Thessaloniki, General Hospital Papageorgiou, Ring Road N. Efkarpia, 56429 Thessaloniki, Greece

## Abstract

*Introduction.* Incisional hernias are a common complication appearing after abdominal wall defects reconstruction, with omphalocele and gastroschisis being the most common etiologies in children. Abdominal closure of these defects represents a real challenge for pediatric surgeons with many surgical techniques and various prosthetic materials being used for this purpose.* Case Report.* We present a case of repair of a postoperative ventral hernia occurring after congenital omphalocele reconstruction in a three-and-a-half-year-old child using an acellular, sterile, porcine dermal mesh.* Conclusion.* Non-cross-linked acellular porcine dermal matrix is an appropriate mesh used for the reconstruction of abdominal wall defects and their postoperative complications like large ventral hernias with success and preventing their recurrence.

## 1. Introduction

Incisional hernias are a common complication appearing after abdominal wall defects reconstruction, with omphalocele and gastroschisis being the most common etiologies in children. Abdominal closure of these defects represents a real challenge for pediatric surgeons with many surgical techniques and various prosthetic materials being used for this purpose.

We present a case of repair of a postoperative ventral hernia occurring after congenital omphalocele reconstruction in a three-and-a-half-year-old child using XenMatrix, an acellular, sterile, porcine dermal mesh.

## 2. Case Presentation

**Figure 1:**
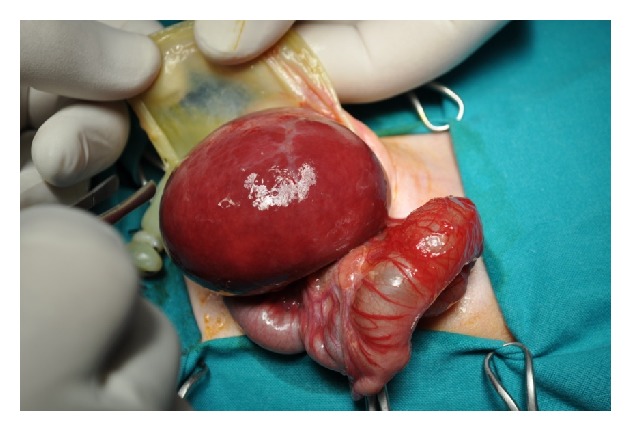
The hernia sac containing the whole liver and part of the large and small intestine.

The female neonate was born after gestational age of 38 weeks and 2 days with an elective caesarian section due to an antenatal diagnosis of congenital omphalocele in the second trimester, with an Apgar score 8 at the 1st minute and 9 at the 5th. This firstborn neonate weighted 2.800 gr at birth and presented a giant omphalocele containing the whole liver and part of the large and small intestine (Figure 1). The rest of the physical examination was normal with no cardiovascular anomalies. The newborn was admitted to the neonatal intensive care unit and was supported with mechanical ventilation using sedation and muscle relaxation for the first 4 days of life. In the second postnatal day the patient underwent the first operation with excision of the hernia sac, complete reduction of the viscera into the peritoneal cavity, and abdominal wall closure without any traction, after identification and separation of the skin from the fascia and debridement of the margins. The early primary closure technique seemed successful and the neonate was in a stable condition postoperatively. An infection was noted on the 7th postoperative day due to colonization of a peripherally inserted central venous catheter with staphylococcus epidermidis. The neonate was dismissed from the pediatric surgery clinic after nursing for a month with no feeding or other problems.

**Figure 2:**
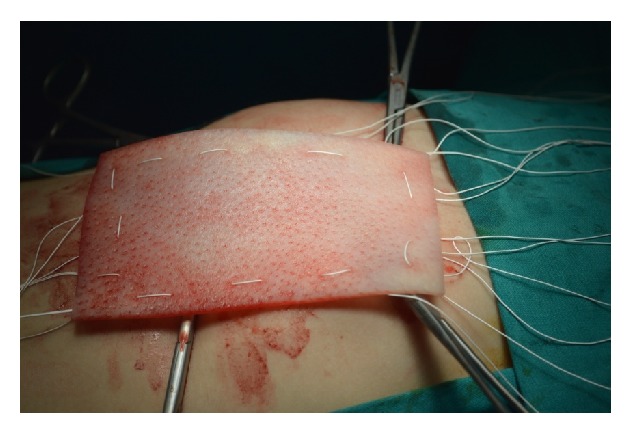
The acellular porcine dermal mesh was cut in a larger size (12 cm × 8 cm) than the ventral hernias' gap dimensions which were 6 cm × 3 cm.

**Figure 3:**
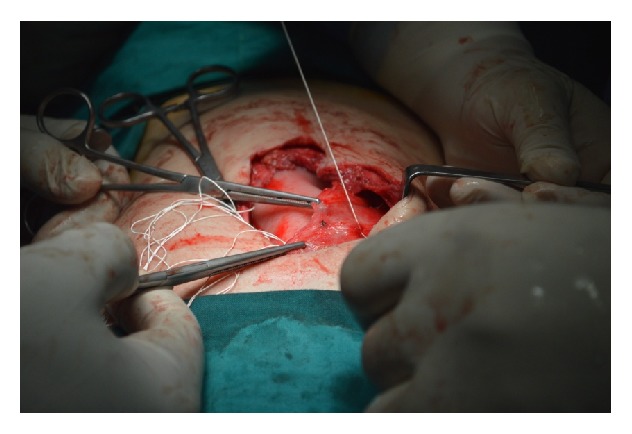
The collagen matrix was positioned in an underlay technique and was secured in the rectus muscles with at least 3 cm overlap beyond the fascial margins using interrupted Gore-Tex 2.0 sutures every 2 cm.

**Figure 4:**
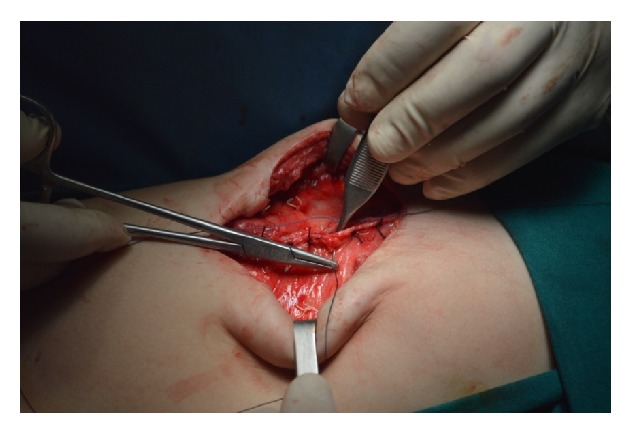
The hypoplastic fascial margins were stitched together, completely covering the mesh according to normal anatomic relations.

At the age of three months the infant underwent plastic surgery of the abdominal wall in order to repair a massive hernia that involved a great part of the anterior abdominal wall due to hypoplasia of the rectus muscles, especially the left one. Unfortunately, ventral hernia reoccurred and at the age of three and a half years the child was operated on for the third time. Under general anesthesia the old scar was removed, the incision was expanded 2 cm under the umbilicus, and, after accessing the peritoneal cavity, excision of adhesions from the previous surgery took place. Rectus muscles were separated from the subcutaneous fat in a distance of 4 cm from the free hypoplastic margins to the midline. The dimensions of the ventral hernia's gap were 6 cm × 3 cm. Porcine biological mesh, an acellular collagen matrix, was positioned in an underlay technique which is thought to result in a longer-term repair. It was cut in a larger size (12 cm × 8 cm) and was secured to the rectus muscles with at least 3 cm overlap beyond the fascial margins using interrupted Gore-Tex 2.0 sutures every 2 cm (Figures 2 and 3). The hypoplastic fascial margins were stitched together, completely covering the mesh (Figure 4). A drainage tube was placed in order to avoid the accumulation of serosanguinous fluid. Finally, abdominal wall closure was established according to normal anatomic relations. After a follow-up period of one year the child remains uncomplicated with a well-structured anterior abdominal wall without evidence of hernia recurrence.

## 3. Discussion

Omphalocele is the most common abdominal wall defect seen in neonates occurring in an incidence of about 2 to 2.5 per 10.000 live births [1]. This midline defect is of variable size and is characterized by extrusion of abdominal viscera, covered by a membranous sac, into the base of the umbilical cord. Failure of the reduction of physiological embryonic umbilical hernia or abnormal development and migration of abdominal wall muscular components are considered the main mechanisms leading to omphalocele.

Management of omphalocele remains a real challenge for pediatric surgeons as difficulties often come up especially in the case of giant omphalocele where there is disproportion between the amount of herniated viscera and the diminished peritoneal cavity. Morbidity and mortality rates are high because this abdominal wall defect is often accompanied by cardiovascular problems and gastrointestinal or chromosomal anomalies.

Early primary abdominal wall closure is the ideal surgical technique for omphalocele management with separate primary repairs of both the fascial and the skin layers. In some cases of giant omphaloceles this approach could result in high intra-abdominal pressure with its known consequences. Many strategies have been used for these cases like Gross's skin mobilization method [2] and Schuster's technique with staged reduction of the extraperitoneal viscera by external pressure applied with sheets of Teflon [3] and its modifications with other materials like silo [4]. Alternatively, tissue expanders placed intraperitoneally or in the abdominal wall can increase the volume of peritoneal cavity and aid in the reconstruction of the abdominal wall defect [5].

In recent years separation component technique, which is often used in adults for reconstruction of large abdominal wall defects, has been applied to pediatric patients too [6]. This technique includes dissection of the abdominal wall subcutaneous tissue from the muscle and fascia and an incision of the external oblique aponeurosis one centimeter lateral to the rectus sheath, allowing medial advancement of the rectus muscles. Sometimes this strategy is accompanied by placement of a prosthetic material in order to minimize tension on final closure, in cases that rectus muscles cannot be fully advanced medially [7].

There is a variety of exogenous meshes that have been used in surgery of abdominal wall defects with greater experience in adults. Prosthetic materials can be biological or synthetic. Polypropylene mesh is the most widely used nonabsorbable synthetic material because of its low cost and its easiness in application [8]. As a foreign body this mesh produces an inflammatory process resulting in scar formation, although for the same reason it can lead to the creation of adhesions, erosions, and fistulas. Comparing the suture repair technique with the open mesh repair, the former is characterized by higher incidence of hernia recurrence, while the latter is related to higher rates of wound infection [9]. The great availability of synthetic devices nowadays makes the decision of selecting the appropriate one for a specific defect very hard to take for the pediatric surgeon. There have been many trials to classify meshes in various categories according to porosity (e.g., macro, microporous, and submicronic pores) or their weight (e.g., ultralight, light, standard, or heavy weight) in an attempt to recognize differences in their properties that could reflect significant changes in clinical outcome [10, 11], although more research is needed for that purpose [9].

An increasing interest for biological matrices is noted recently, considering the limitations of synthetic meshes. Biological scaffolds are materials composed of extracellular matrix derived from a variety of tissues of human or other mammalian origins. Their utility leans on their ability to interact with the host tissue and facilitate its regeneration by undergoing revascularization and remodeling, although maintaining their structural integrity and disinclining fibrotic tissue formation [12]. Biological meshes are biodegradable, unless processes like cross-linking the collagen fibers take place. In such a case the implant is thought to be more stable by preventing its breakdown from collagenase, although there is an increased possibility for inflammation and graft destruction [13].

XenMatrix is a sterile, acellular, non-cross-linked porcine dermal matrix. AquaPure process, which is characterized by tissue harvesting, cleaning, cellular material removal, viral inactivation, and e-beam sterilization, results in an effective cell abolishment while maintaining the graft's structure and strength. This collagen scaffold allows early cellular infiltration, incorporation in the host tissue, and revascularization, without a significant loss of strength during the healing period which is critical for the abdominal wall reconstruction. Another advantage of this biological mesh is the broad range of available sizes giving the opportunity to the surgeon for repairing quite large defects (18 sizes, the greatest 30 × 45 cm, and thickness from 1.8 to 2.5 mm). Moreover it does not need reconstitution and it is ready for implanting immediately.

XenMatrix has been used in the reconstruction of complex abdominal wall defects in adults with success and it seems effective and safe, with good tolerance and limited complications [14–17]. Seroma, infection, inflammation, allergy, adhesion, fistula formation, hematoma, and recurrence of tissue defect are the potential adverse reactions seen with the use of any prosthesis. Synthetic meshes present limited tissue incorporation and greater percentages of adhesions, erosions, or fistula formations especially in cases of infection, while they need removal once contaminated [18]. Comparatively with human derived acellular dermis matrices, XenMatrix is less expensive, sterile, and easier to use due to its availability in larger sizes [14]. Moreover elastic fibers existing in human grafts can be responsible for their laxity under circumstances of stretch leading to hernia recurrence [19].

Experience of surgical management of omphalocele using biological products in pediatric population is limited without the ability of comparing the clinical outcome according to the selected material for the operation. There are some small series of neonates that underwent abdominal wall reconstruction with the use of human acellular dermis (Alloderm) with a desirable outcome [20].

Porcine small intestine submucosa (Surgisis) is the most used biological product in the management of pediatric abdominal wall defects in recent years. The rates of recurrent herniation seem to be similar regardless of the prosthetic material used. Grethel et al. performed a retrospective review of 152 cases of newborns with congenital diaphragmatic hernia. Twelve (44%) of 27 patients who underwent repair with Surgisis presented recurrent herniation comparatively with 17 (38%) of 45 neonates who had a Gore-Tex repair. The time to recurrence was similar in both groups with most recurrences occurring in the first year [21]. St Peter et al. investigated 81 survivors from congenital diaphragmatic hernia, 24 operated on with a synthetic patch and 57 with primary repair. Those repaired with a patch exhibited a significantly increased risk of recurrence, small bowel obstruction, and subsequent operation. Eleven patients had nonabsorbable meshes and 13 were repaired with absorbable ones (Surgisis). While there were no differences in recurrence between these two groups, four patients (31%) with Surgisis developed small bowel obstruction compared with the one patient (9%) repaired with a nonabsorbable synthetic [22]. In another case series of 22 neonates repaired with a primary patch (13, Surgisis, and 9, polytetrafluoroethylene), there was a similar recurrence rate in the two groups (31% and 33%, resp.) [23]. Beres et al. evaluated 13 patients with different abdominal wall defects who were repaired with Surgisis (2 with gastroschisis, 2 with ventral hernia after diaphragmatic hernia repair, and 9 with omphalocele). At a median follow-up of 60 months, there was a recurrence rate of 46% with a trend toward infantile age [24]. Naji et al. report their experience with the implantation of Surgisis in such defects with encouraging results. From the 24 pediatric patients 4 developed seroma, 2 had wound infection, and there were 2 recurrences with incisional hernia formation which resolved though spontaneously [25].

Another bioactive material used in the repair of abdominal wall defects is Permacol, a cross-linked acellular porcine dermal collagen mesh. Mitchell et al. reported no recurrences in patients treated with Permacol comparatively with 28% recurrence rate in those repaired with Gore-Tex in a case series of 37 neonates who underwent a patch repair of congenital diaphragmatic hernia [26]. Moreover, there is a case report with the application of Permacol for a traumatic abdominal wall repair in an 8-year-old boy with a favorable outcome [27].

An uncomplicated postoperative outcome has also been reported from Caso Maestro et al. in a series of six pediatric patients who underwent a delayed abdominal wall closure after liver transplantation, as primary closure was impossible due to a size mismatch between the large graft and the small recipient. A non-cross-linked porcine-derived acellular dermal matrix (Strattice) was used for the abdominal wall repair in all cases. After a mean follow-up of 26 months, no recurrences or other complications were noted in these vulnerable patients [28].

We report our case because we succeeded to manage a large ventral hernia, which is a common complication of abdominal wall defect reconstruction, with the use of a non-cross-linked acellular dermal matrix in a child with a favorable outcome. Further studies are needed in order to clarify its role in the repair of abdominal wall defects and to estimate the possibility of adverse surgical outcomes.
